# Expression of a type B RIFIN in *Plasmodium falciparum* merozoites and gametes

**DOI:** 10.1186/1475-2875-11-429

**Published:** 2012-12-21

**Authors:** Steven B Mwakalinga, Christian W Wang, Dominique C Bengtsson, Louise Turner, Bismarck Dinko, John P Lusingu, David E Arnot, Colin J Sutherland, Thor G Theander, Thomas Lavstsen

**Affiliations:** 1 Centre for Medical Parasitology, Department of International Health, Immunology, and Microbiology, University of Copenhagen and at Department of Infectious Diseases, Copenhagen University Hospital (Rigshospitalet), 1014, Copenhagen, Denmark; 2 Department of Infectious and Tropical Diseases, London School of Hygiene and Tropical Medicine, London, UK; 3 National Institute for Medical Research (NIMR), Tanga Medical Research Centre, Tanga, Tanzania; 4 Institute of Immunology and Infection Research, School of Biology, University of Edinburgh, West Mains Road, Edinburgh, EH9 3JT, Scotland, UK

## Abstract

**Background:**

The ability of *Plasmodium falciparum* to undergo antigenic variation, by switching expression among protein variants encoded by multigene families, such as *var*, *rif* and *stevor*, is key to the survival of this parasite in the human host. The RIFIN protein family can be divided into A and B types based on the presence or absence of a 25 amino acid motif in the semi-conserved domain. A particular type B RIFIN, PF13_0006, has previously been shown to be strongly transcribed in the asexual and sexual stages of *P. falciparum in vitro*.

**Methods:**

Antibodies to recombinant PF13_0006 RIFIN were used in immunofluorescence and confocal imaging of 3D7 parasites throughout the asexual reproduction and sexual development to examine the expression of PF13_0006. Furthermore, reactivity to recombinant PF13_0006 was measured in plasma samples collected from individuals from both East and West African endemic areas.

**Results:**

The PF13_0006 RIFIN variant appeared expressed by both released merozoites and gametes after emergence. 7.4% and 12.1% of individuals from East and West African endemic areas, respectively, carry plasma antibodies that recognize recombinant PF13_0006, where the antibody responses were more common among older children.

**Conclusions:**

The stage specificity of PF13_0006 suggests that the diversity of RIFIN variants has evolved to provide multiple specialized functions in different stages of the parasite life cycle. These data also suggest that RIFIN variants antigenically similar to PF13_0006 occur in African parasite populations.

## Background

The life cycle of the *Plasmodium falciparum* malaria parasite involves asexual and sexual phases. To maintain a persistent infection in the human host for successful transmission to mosquitoes, parasites express various polymorphic proteins that help evade human antibody responses and facilitate invasion of host cells. During asexual multiplication in the blood, parasites invade and multiply inside erythrocytes, apart from short periods as extracellular merozoites, which are released at erythrocyte rupture and then quickly re-invade fresh host cells. Polymorphic proteins like merozoite surface proteins 1 (MSP-1) and apical membrane antigen 1 (AMA-1) [1,2] are expressed on the merozoite surface and are known to play specific roles in erythrocyte invasion. The STEVOR family of variant antigens are also known to be expressed on the merozoite surface [3] and to be associated with the plasma membrane of mature gametocyte-infected erythrocytes [4]. The locations of the related, highly diverse RIFIN antigen family members are less well understood, but they have been reported to be present inside the merozoite [5]. Each parasite carries approximately 150–200 *rif* and 30–35 *stevor* gene copies per genome, and it remains a possibility that their abundance and diversity also contribute to immune evasion by merozoites during their brief extra-cellular phase.

While it is uncertain whether *rif* genes are expressed in a ‘relaxed’ or strictly mutually exclusive manner, multiple RIFIN variants have been reported in bulk cultures of parasites grown *in vitro*[6,7]. Rifin variants can be divided into A- and B-types based on the presence or absence of a 25 amino acid motif in the semi-conserved domain [8] and sub-structuring of RIFIIN protein sequence reflect functional divergence with A- and B-types serving different roles in distinct parasite stages [5]. During intraerythrocytic multiplication B-type RIFIIN were reported to be retained inside the parasites while A type RIFIN were expressed on the infected erythrocyte surface, potentially contributing to the antigenic variation capacity of the parasite [5].

*Plasmodium falciparum* pathology is profoundly influenced by the sequestration of infected erythrocytes to microvascular endothelium in various tissues. This involves interactions between parasite adhesins and several human endothelial receptors including CD36, ICAM1 and the glycosaminoglycan, CSA [9,10]. During sexual development *in vivo,* mature gametocytes of *P. falciparum* (Stage V) do not appear in the peripheral blood circulation until 7–15 days after the initial wave of blood stream infection appears [11]. This is due to the sequestration of immature gametocyte forms, which develop in various host tissues including the bone marrow and spleen [12,13]. Although superficially analogous to the sequestration of mature asexual parasite stages, the details of interactions between developmental stages of gametocytes and host tissues are poorly understood, and if cytoadherence is involved, the host receptors responsible remain unidentified. Candidate receptors for adhesion of early gametocytes (Stage I, II) include CD36 [14] and for stage III to IV include ICAM-1, CD49c, CD164 and CD166 [15]. Candidate gametocyte-expressed parasite ligands may include variants of the multigene families *var, rif* and *stevor*. Of these, cytoadhesive properties have only been demonstrated for PfEMP1, which has been linked to cytoadhesion of gametocyte stages I to IIA. In the later stages III to IV, PfEMP1 was observed to be retained inside parasite cytoplasm [16]; possibly indicating that PfEMP1 may not be involved in gametocyte cytoadherence after stage IIB. However, more recent transcriptional data suggest that certain group C *var* genes are selectively transcribed during gametocytogenesis *in vitro*[17], suggesting a role for this subset of PfEMP1 in gametocytes, gametes or later parasite stages in the mosquito. Type A RIFIN has been found on the surface of developing gametocytes and type B Rifin expressed but retained inside the cell at all gametocyte stages [18]. STEVOR proteins are localized near the developing gametocyte surface membrane, but surface exposure and any direct role in adhesion to host tissues, remains to be confirmed [4]. However, recently, Tibùrcio and colleagues [19] showed that cell rigidity of immature gametocyte-infected erythrocytes was associated with the expression of STEVOR proteins, potentially contributing to the sequestration of these stages by mechanical retention rather than adhesion [20].

Although antisera have been developed which can distinguish type A from type B RIFIN sub-groups [18], variant-specific RIFIN antibodies have not previously been described. In previous studies [18,21] global transcription analysis of all *rif* genes in sexual and asexual development of the 3D7 parasite line revealed a unique expression pattern of the type B *rif* gene PF13_0006. This gene was up-regulated in late stage schizonts, developing gametocytes and in sporozoites. To test the hypothesis that this RIFIN variant has a distinctive role in parasite development, antibodies to the protein encoded by PF13_0006 were developed and the expression was followed throughout parasite development *in vitro*. Evidence is presented suggesting that this specific type B RIFIN variant is expressed on the surface of free merozoites, internally in developing gametocytes and on the surface of gametes at the point of emerging from activated, mature stage V gametocytes.

## Methods

### Sequences, alignments and distance tree analysis

The variable domain, V2, of 3D7, HB3 and DD2 RIFIN amino acid sequences were aligned, viewed and Neighbour Joining distance trees built as previously described [22]. To these earlier described sequences [22] 107 IT/FCR3 RIFIN sequences retrieved by text search in http://www.plasmoDB.org[23] were included in the alignments giving a total of 481 RIFIN amino acid sequences. Sequence logos were generated using WebLogo (version 2.8) [24].

### Parasite culture and synchronization

Asexual blood stages of *P. falciparum* parasites of the line 3D7 were cultured and magnet-purified based on standard protocols, with modifications as previously described [17,25-27]. Gametocytes of *P. falciparum* clone 3D7 were cultivated using previously established methods [28-31]. Culture media was changed on a daily basis and parasites were monitored by visualizing of Giemsa-stained smears under the light microscope. Parasites at various develo7pmental stages of gametocytogenesis were harvested and purified using MACS as described previously [32,33]. To activate mature stage V gametocytes to differentiate into gametes, parasites were incubated for 10 minutes at room temperature with five times pellet volume of 100 μM Xanthurenic acid (Sigma-Aldrich) in cold RPMI. Parasites were then harvested and observed microscopically during rounding up and exflagellation.

### Recombinant protein and antibody production

Recombinant proteins were produced in the baculovirus expression system as described previously [34]. Primer pairs were designed to amplify the variable domain of *PF13_0006* and *PFD0070c rif* genes from genomic DNA of the 3D7 parasite line. PCR products were cloned into baculovirus expression vector pAcG2T (BD Bioscience) containing an N-terminal GST tag, and expressed as previously described [35]. Recombinant proteins were harvested and purified on gluthatione sepharose columns. Polyclonal antisera were generated in rabbits as previously described [36]. Experiments including immunizations and bleeding of animals were approved by The Danish Animal Procedures Committee (“Dyreforsoegstilsynet”) as described in permit no. 2008/561-1498 and according to the guidelines described in act no. LBK 1306 (23/11/2007) and BEK 1273 (12/12/2005). The antiserum was tested positive in ELISA for reactivity against the immunizing antigen and the antiserum was depleted for antibodies reacting with erythrocyte antigens by mixing equal amounts of antiserum with human O^+^ erythrocytes and incubating 24 h at 4°C. The depleted antiserum was IgG purified on protein G sepharose columns and dialyzed overnight in PBS. To demonstrate the specificity of the anti-PF13_0006 IgG, IT/FCR3 parasites, which do not contain a *rif* with high sequence similarity to PF13_0006 in their genome, were used in immunofluorescence and western blot analyses (Additional file 1 and Additional file 2, respectively). As a negative control for the anti-PF13_0006 IgG preparation, a rabbit with a recombinant protein representing another RIFIN (PFD0070c) was immunized. The resulting anti-PFD0070c antibody did not react with parasite extracts, either because the protein was not expressed by the cultured parasites or because the antibodies did not react with the native protein. However, these antibodies did show ELISA reactivity against the recombinant protein used for the immunization and was hereafter termed as control IgG.

### Primer design and quantitative PCR

RNA extraction and quantitative reverse-transcriptase PCR were carried out as previously described [17,21]. Quantitative primers for *rif* gene *PF13_0006*, universal *rifA2* and for amplification of the endogenous comparator genes *seryl tRNA synthetase* and *fructose-bisphosphate aldolase*, have been described previously [22,37]. Primers were designed for *rif* subgroups *RifA1* and *RifA3*[20]: *RifA1* F 5 ACAAAATTTGGGAGGGGTTG 3 and *RifA1* R 5 TGCCTTAAGACCTGCATCTG 3; and *RifA3* F 5 AGCAGGACATTTGGCRGGTAC 3 and *RifA3* R 5 ACKAGGTGTACATRACGTGR 3.

### Immunofluorescence assays

#### IFA on fixed smears

Immunofluorescent antibody assays were performed on gametocytes and asexual stages on fixed smears using standard methods [34]. Primary antibodies used were rabbit IgG raised to PF13_0006 and control IgG, monoclonal antibody 1H12 specific for Pfg27/25 [38] and monoclonal antibody against Pfs230 [39]. Visualization was with the 60X oil immersion lens on a Nikon TE 2000-E confocal microscope.

For the analysis of gametes, fixation was carried out without permeabilizing the parasites with Triton X-100. An antibody recognizing human glycophorin A (BD Bioscience) was used to visualize the erythrocyte membrane.

#### Double immunofluorescent detection of PF13_0006 and MSP-1_19_ in live schizonts

Synchronized live schizonts were MACS purified in warmed 2% BSA/FCS and permeabilized at 30°C with 500 μl pneumolysin at a 300 μg/ml concentration. The perforated schizonts were washed twice with 1% PBS/BSA resuspended in 92 μl 1% PBS/BSA, 4 μl PF13_0006 or 4 μl control IgG, 1 μl MSP-1_19_[40] antibodies and 3 μl DAPI at 5 μg/ml. The parasites were incubated for 45 minutes at 4°C, washed three times with 1% PBS/BSA and then incubated for 30 minutes at 4°C with 100 μl Alexa 488 anti-rabbit/alexa 568 anti-mouse IgG (Invitrogen) at 1/2000 and 3 μl DAPI (Sigma) at 5 μg/ml. After thorough washing, schizonts were vizualized on live wet preparation with laser scanning confocal microscopy performed using a Nikon TE 2000-E confocal microscope with 60X oil immersion objective lens (DCI) as previously described [41].

#### Double immunofluorescent detection of PF13_0006 and MSP-1_19_ on live merozoites

Merozoites were collected according to a previous method [42] with some modifications. Briefly, Concanavalin (5 mg/ml) in sterile distilled water was added to cover the bottom of a sterile petri plate (falcon) and incubated at 37°C for 30 minutes. The plate was then washed with distilled water. A synchronized parasite culture at schizont stage was added to the plate and incubated for 30 minutes. The plate was carefully washed with RPMI media and new media was added. The parasites were incubated for two hours at 37°C. The supernatant containing the merozoites was collected, centrifuged at 2,000 rpm for five minutes, washed three times in 1% PBS/BSA and then stained as above. Live schizonts and merozoites of the parasite line IT/FCR3 were used as controls and were stained as above with following modification: no double staining was done and only Alexa 488 anti-rabbit IgG (Invitrogen) was used. The colour of the MSP-1_19_ staining was converted using Nikon EZ-C1 Freeviewer Software from green to red for easy comparison with the staining of the 3D7 parasite line.

### Western blot

Protein extracts from MACS-purified mature stage gametocytes from day 15 of gametocytogenesis were further concentrated using a modified two detergent protocol comprising two cold 1% Triton X-100 extractions followed by 2% sodium dodecyl sulphate (SDS) extraction. For western blotting, protein extracts were mixed with loading dye and separated by SDS-PAGE before being transferred to nitrocellulose membrane for two hours at 30 V. Proteins of interest were visualized by sequential incubation with primary and secondary antibodies followed by chemiluminescent detection.

### Serological analyses

Plasma samples were obtained during village surveys as part of on-going monitoring of malaria endemicity on North Eastern Tanzania [43]. For this study, 1,303 samples from children and adults aged six months to 60 years were thawed and analyzed for presence of IgG reacting with recombinant PF13_0006. Plasma samples from children participating in malaria drug efficacy trials in The Gambia [33] were also tested. The antibody reactivity was measured in a bead based Luminex assay as previously described [44], using beads coated with the PF13_0006 and control beads coated with GST. For each donor the reactivity with GST coated beads was subtracted from the reactivity measured with beads coated with PF13_0006. Cut-off was based on the mean reactivity +2 SD of European donors never exposed to malaria. Informed consent forms were signed by parent or legal guardians of all subjects and the trials in Tanzania and in The Gambia were reviewed and approved by the Medical Research Coordinating Committee, Tanzania (NIMR/HQ/R.8a/Vol.IX/559), and by the Medical Research Council/Gambian Government Joint Ethical Committee, respectively.

## Results

### PF13_0006 RIFIN is expressed in the schizonts but not in the ring stages of asexual parasites

**Figure 1:**
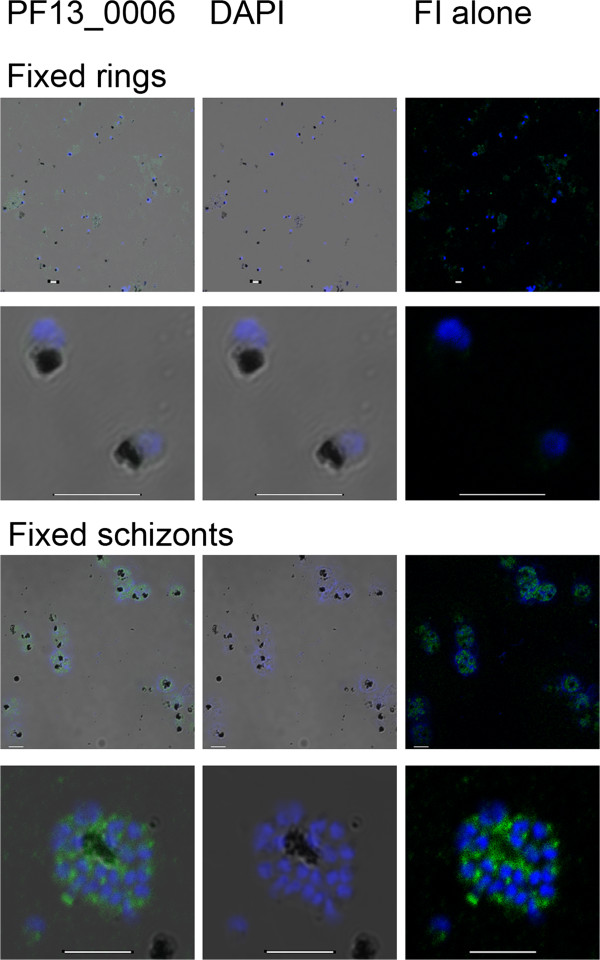
**Immunofluorescence analysis of PF13_0006 expression in fixed asexual *Plasmodium falciparum *parasites.** Ring stage parasites (first two rows) and schizonts (last rows) of the 3D7 line were fixed, permeabilized, and analyzed by staining with rabbit anti-PF13_0006 IgG (green). Nuclei were stained with DAPI (blue). DIC shadow-cast images with the fluorescence image superimposed in the first two coloumns and the fluorescence image (FI) alone in the last coloumn. Scale bar 5 μM.

Asexual *P. falciparum* parasites of the 3D7 line were synchronized and rabbit IgG raised to the variable domain of PF13_0006 RIFIN was used to detect the expression of this protein in fixed permeabilized smears of ring and schizont parasites. In agreement with previously published *rif* transcript analyses, no RIFIN protein was detected in the ring stages (Figure 1 first and second row), whereas the proportion of anti-PF13_0006 IgG stained schizonts was higher than 90% (Figure 1 third and fourth row). The staining showed that PF13_0006 is retained inside the infected erythrocytes. The expression of PF13_0006 was confirmed by western blot, where a correct sized band in 3D7 schizonts and not in 3D7 ring and FCR3 schizonts extracts was observed (Additional file 2). Furthermore, higher abundance of PF13_0006 transcript in schizonts compared to ring stage parasites was confirmed by quantitative PCR (Additional file 3).

### PF13_0006 RIFIN expression of live merozoites

**Figure 2:**
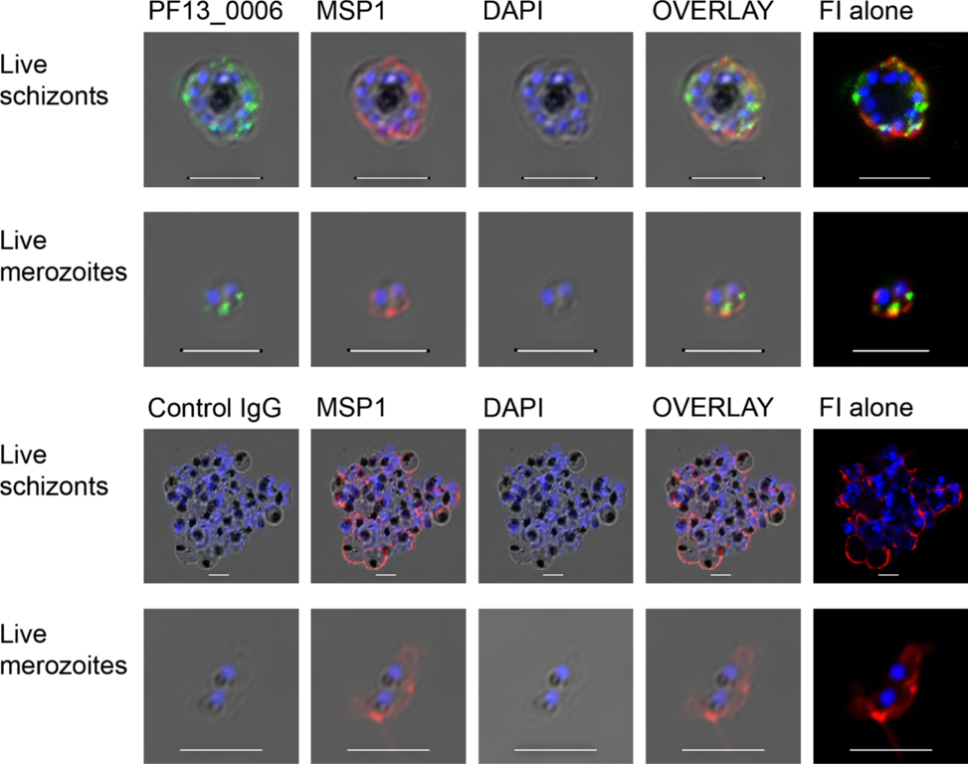
**Immunofluorescence analysis of PF13_0006 expression in live asexual *Plasmodium falciparum *parasites.** Live schizonts (first row) and merozoites (second row) of the 3D7 parasite line were analyzed by staining with anti-PF13_0006 IgG (green) and anti-MSP-1 Ab (red). As a control, live schizonts (third row) and live merozoites (fourth row) were stained with control IgG (green) and anti-MSP-1 Ab (Red). Nuclei were stained with DAPI (blue). DIC shadow-cast images with the fluorescence image superimposed in the first four coloumns and the fluorescence image (FI) alone in the last coloumn to augment the visualisation of the staining. Scale bar 5 μM.

To investigate if the PF13_0006 RIFIN was expressed on the surface of the developing merozoites, live schizonts were permeabilized with pneumolysin to allow the passage of antibodies through the membrane of the infected erythrocyte. Co-localisation on merozoites was examined by double staining with monoclonal antibodies directed against MSP-1_19_ and either Rifin antibodies, anti-PF13_0006 IgG or control IgG. Interestingly, both anti-PF13_0006 IgG and anti-MSP-1_19_ Abs stained the dividing merozoites inside the infected erythrocyte (Figure 2, first row and Additional file 4). Similar staining was performed on free, non-permeablized, non-fixed live merozoites. Anti-PF13_0006 IgG stained the surface of the merozoites and its location appeared to overlap with MSP-1_19_ (Figure 2, second row and Additional file 4). No staining of schizonts or merozoites was observed with the control IgG (Figure 2, third and fourth row). In a representative experiment, eight of 10 free merozoites stained with anti-PF13_0006 IgG. The analyses were repeated on the IT/FCR3 parasite. IT/FCR3 does not contain a *rif* with high sequence similarity to PF13_0006 in its genome and as expected no staining of schizonts or merozoites was observed (Additional file 1).

### PF13_0006 RIFIN is expressed throughout gametocyte development

**Figure 3:**
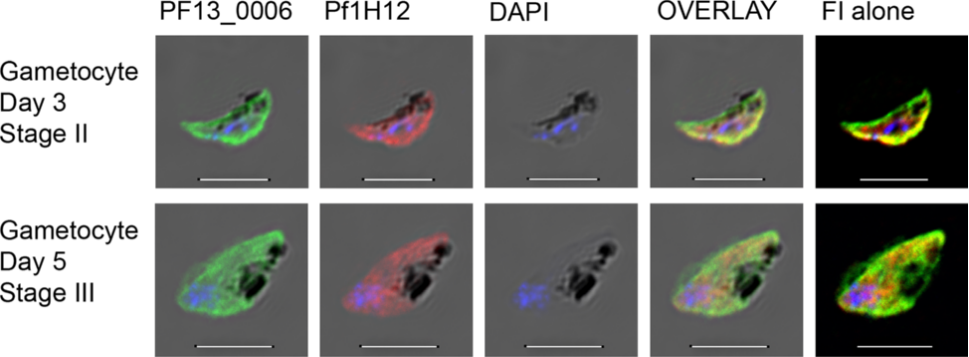
**Immunofluorescence analysis of the PF13_0006 expression in fixed permeablized early stage gametocytes.** Gametocytes of the *P. falciparum* 3D7 line stage II (first row) and stage III (second row) were stained with anti-PF13_0006 IgG (green) and 1H12 (anti-Pfg27/25) (red). Nuclei were stained with DAPI (blue). DIC shadow-cast images with the fluorescence image superimposed in the first four coloumns and the fluorescence image (FI) alone in the last coloumn. Scale bar 5 μM.

**Figure 4:**
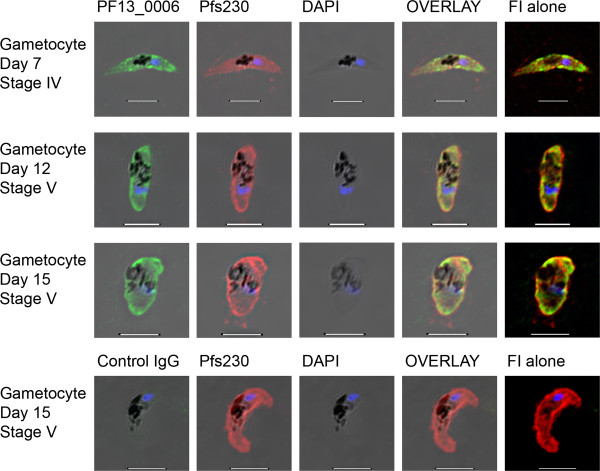
**Immunofluorescence analysis of the PF13_0006 expression in fixed permeablized late stage gametocytes.** Gametocytes of the *P. falciparum* 3D7 line at day 7 stage IV (first row), day 12 stage V (second row), and day 15 stage V (third row) were stained with anti-PF13_0006 IgG (green) and anti-Pfs230 Ab (red). As a control, gametocytes at day 15 stage V (fourth row) were also stained with control IgG (green) and anti-Pfs230 Ab (red). Nuclei were stained with DAPI (blue). DIC shadow-cast images with the fluorescence image superimposed in the first four coloumns and the fluorescence image (FI) alone in the last coloumn. Scale bar 5 μM.

The sub-cellular localization of PF13_0006 protein was followed throughout gametocyte development from stages I to stage V. Parasites were tested live and intact or after formaldehyde-fixation and Triton X-100-permeabilization in parallel to define proteins expressed on the surface of the infected erythrocytes and protein expressed in intracellular compartments. To distinguish gametocytes at different stages of development, parasites were stained using an early stage specific monoclonal antibody, 1H12, recognizing Pfg27/25 [38] (Figure 3) and a late stage specific monoclonal antibody, recognizing Pfs230 [39] (Figure 4). Neither the anti-PF13_0006 IgG nor control IgG stained any of the live gametocyte stages I-V. However, the analyses of fixed, permeablized preparations revealed expression of PF13_0006 inside the gametocytes of all stages (Figures 3 and 4). The proportion of gametocytes stained with the anti-PF13_0006 IgG increased from 65% during gametocyte maturation reaching 78% for stage V, day 15 parasites.

### Anti-PF13_0006 IgG detects protein of similar size to PF13_0006 Rifin in western blots of mature stage V gametocyte protein extracts

**Figure 5:**
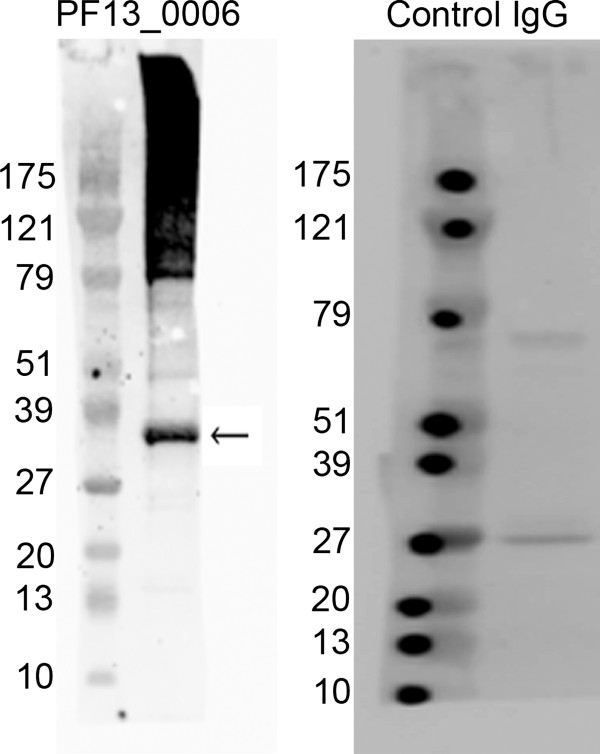
**Western blot for PF13_0006 expression in mature stage V gametocytes.** Protein extracts were obtained from day 15 mature stage V gametocytes and the expression of PF13_0006 RIFIN was analyzed by western blot. A band of approximately 38 kDa was detected by anti-PF13_0006 IgG (left panel, arrow). Control IgG showed two bands of approximately 27 and 78 kDa (right panel).

Proteins were extracted from day 15 mature stage V gametocytes for western blot analysis. The anti-PF13_0006 IgG detected a protein band of similar size to the predicted molecular weight (37.8 kDa) of PF13_0006 (Figure 5), indicating that full length PF13_0006 RIFIN is expressed in mature stage V gametocytes. Also bands of approximately 79 kDa and larger were detected of unknown origin. Similarly, protein band smear above 79 kDa was observed in western blot of FCR3 schizonts (Additional file 2); however, FCR3 schizonts were negative in confocal microscopy (Additional file 1), indicating that the +79 kDa proteins detected here are not detected by confocal microscopy in 3D7 gametocytes. Western blots using control IgG detected two bands of approximately 27 kDa and 78 kDa as also seen for the 3D7 schizonts (Additional file 2).

### PF13_0006 RIFIN expression of gametes

**Figure 6:**
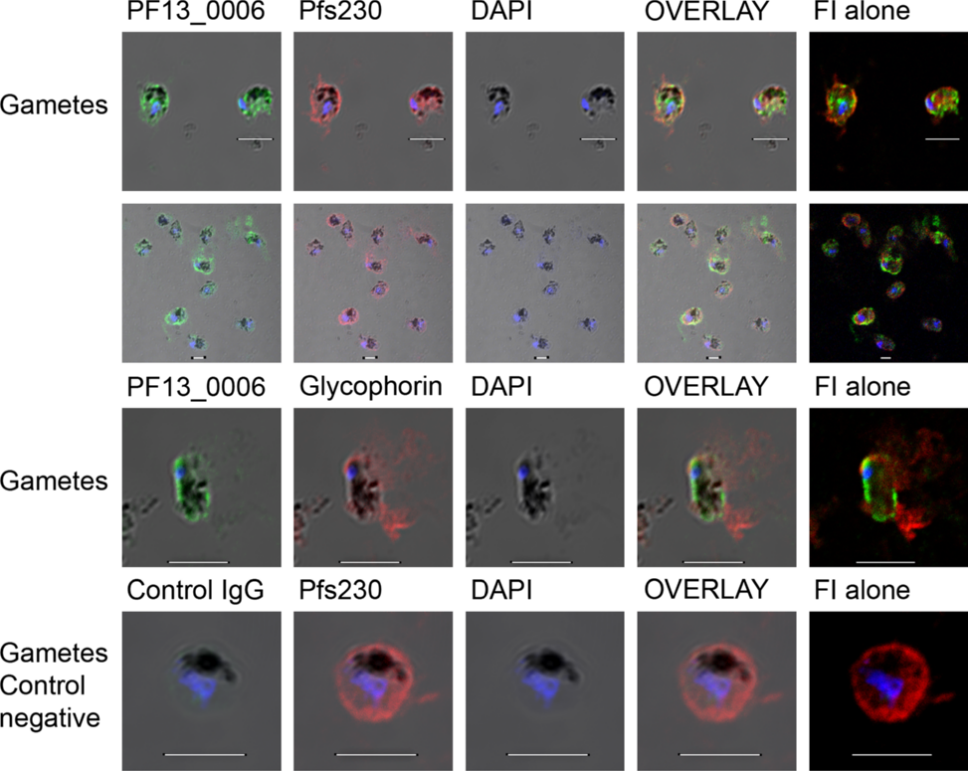
**Expression of PF13_0006 on the surface of activated gametocytes (gametes).** Activated gametocytes (emerging gametes) of the *P. falciparum* 3D7 line were fixed but not permeabilized and stained with anti-PF13_0006 IgG (green) and anti-Pfs230 Ab (red) (first two rows) and anti-PF13_0006 IgG (green) and anti-glycophorin A (red) (third row). As a control gametes were stained with control IgG (green) and anti-Pfs230 Ab (red). Nuclei were stained with DAPI (blue). DIC shadow-cast images with the fluorescence image superimposed in the first four coloumns and the fluorescence image (FI) alone in the last coloumn. Scale bar 5 μM.

Mature gametocytes from day 15 were activated for differentiation into gametes by incubation with xanthurenic acid at room temperature. Activated gametocytes lose their crescent shape and become round gametes before escaping from the erythrocyte membrane [45]. Initially, a double staining indirect immunofluorescence assay was done on formaldehyde-fixed gamete smears using anti-PF13_0006 IgG and antibodies recognizing the gamete antigen Pfs230, for confirmation of the parasite stage (Figure 6 first and second row). The surface of the rounded parasites was observed to be stained with both antibodies and the proportion of anti-PF13_0006 IgG stained parasites was higher than 90%. In order to confirm the presence of RIFIN on the surface of the gamete and not the erythrocyte membrane, an experiment was conducted to investigate the integrity of the erythrocyte cell membrane. An antibody specific for the human erythrocyte cell membrane protein, glycophorin A, was used for double staining with anti-PF13_0006 IgG. Interestingly, anti-PF13_0006 IgG stained the activated gametes whereas the glycophorin A appeared detached from the parasite surface (Figure 6, third row and Additional file 5). This supports surface-expression of PF13_0006 on emergent gametes released from the host erythrocyte. Determination of the sexes of the gametes was not established and thus whether the PF13_0006 expression is sex-specific is not known.

### Sequence analysis of B1 RIFINs

Previous grouping of the variable domain, V2, of RIFINs [8] has identified group A, B and subgroups B1 and B2 RIFINs. PF13_0006 is one of 37 RIFINs grouped as a B1 RIFIN in parasite clones 3D7, HB3, DD2, and IT/FCR3 [22]. To further identify sequence traits characteristic for the B1 RIFINs, the V2 of 481 3D7, IT/FCR3, HB3 and DD2 RIFIN sequences was split in three, V2-A, V2-B and V2-C, based on two central cysteines present in most sequences (~88%) (Additional file 6A). The three V2-subdomains were re-aligned separately and Neighbour Joining distance trees were built showing the V2-C domain being the best to cluster the sub-group B1 RIFINs (Additional file 6B). A sequence logo was generated based on the V2-C domain of 37 B1 RIFINs showing that the C-terminal part of the V2-C is particularly conserved and unique for the B1 RIFINs (Additional file 6C). The average sequence identity of this part is 62%.

### Reactivity against PF13_0006 in plasma from malaria endemic areas

After identifying a relatively conserved sequence motif in the hypervariable V2 domain of B1 RIFINs, the sero-prevalence of IgG antibodies to recombinantPF13_0006 was evaluated among 1303 malaria-exposed individuals living in North Eastern Tanzania. In this group of individuals the prevalence of antibodies in peripheral plasma that were able to recognize recombinant PF13_0006 was 7.4% (Additional file 7A). Multiple logistic regression analysis demonstrated that the presence of antibodies was significantly more common among children over five years than in either those below five years or in adults. Despite Mkokola village having significantly higher transmission than Kwamasimba village, recognition of PF13_0006 was not significantly more common among individuals from Mkokola. Despite a progressive decline in malaria transmission in the two villages, these antibodies were most common in samples collected in 2008 (Table 1).

**Table 1:** Anti-PF13_0006 IgG antibodies among 1303 individuals living in North Eastern Tanzania

	**Odds ratio and 95% confidence interval**	**P value**
Age group (years)		
0-2 (N = 172)	1 (reference group)	
2-4 (N = 370)	2.9 [0.8-9.9]	0.093
4-7 (N = 492)	7.8 [2.4-25.3]	0.001
7-11 (N = 309)	7.7 [2.3-25.2]	0.001
12-60 (N = 180)	3.7 [1.0-13.4]	0.049
Year of sampling		
2004	1 (reference)	
2005	1.4 [0.7-2.9]	0.37
2006	0.9 [0.4-2.1]	0.83
2007	0.7 [0.3-1.6]	0.43
2008	2.4 [1.2-4.6]	0.011
2009	0.8 [0.4-4.6]	0.64
Village		
Kwamasimba (Low transmission village)	1 (reference)	
Mkokola (High transmission village)	1.1 [0.7-1.7]	0.53

Multiple logistic regression model showing odds ratios. Samples were collected from two villages during annual surveys from 2004–2009.

Furthermore, plasma samples from 182 Gambian children participating in malaria drug efficacy trials during 2001 and 2002 [33] were also evaluated for the presence of antibodies recognizing recombinant PF13_0006. The seropositivity rate was 12.1% (Additional file 7B).

## Discussion

The *P. falciparum* parasite uses clonal antigenic variation to evade host antibody immune responses and permit the establishment of the fairly long-lasting infections that individual clones require for their transmission back to the mosquito vector. It is difficult to know what the average clonal transit time through the human host is, but it is unlikely to be shorter than three weeks and could be significantly longer. While the important role of PfEMP1 in antigenic variation during the intra-erythrocytic multiplication stages is well established, antigenic variation during merozoite, gametocyte and gamete stages has not been formally demonstrated in *P. falciparum*, although a role for RIFIN and STEVOR proteins remains plausible.

A-type Rifins are found associated with the surface of infected erythrocytes and gametocytes and thus appear to follow the expression pattern of PfEMP1 [5], whereas surface exposure of STEVOR so far has only been confirmed on merozoites [3]. The data presented here show that a specific type B RIFIN variant (PF13_0006) is expressed by free, live merozoites and gametes (activated gametocytes). Transcript analysis presented here and previously [21,22] unambiguously show that this RIFIN is upregulated in early ring, schizonts and late stage gametocytes. To investigate PF13_0006 protein expression, rabbit antibodies to the variable region of this protein were generated. These antibodies stained the surface of 3D7 merozoites and gametes, but not the surface of intact 3D7 gametocytes or FCR3 merozoites. Also the antibodies only detected correctly sized protein in western blots of 3D7 schizonts and gametocytes extracts and not of 3D7 rings or FCR3 schizonts. Thus, although the antibodies appear to un-specifically target a number of other proteins in the western blots, collectively the data support the expression of PF13_0006 protein on the surface of merozoites and gametes. The expression of PF13_0006 inside the parasite during schizont and gametocyte stages is in agreement with previous reports using antibodies with specificity for B-type RIFINs [5,18]. There is as yet no evidence that expression of RIFIN variants is mutually exclusive [5,36]. Transcript analyses published earlier [21,22] indicate that transcripts of other B1 RIFINs, in particular PFI0025c, encoding the unique sequence motif identified in this study are present in merozoites and gametes at the same time as PF13_0006. High transcript levels of *PF13_0006* were also detected in early rings [22] but no protein was detected which could reflect a spill-over of late stages in the synchronization. It should also be noted that the method for detecting transcripts is much more sensitive than the methods used to detect protein.

A proportion of malaria exposed individuals from both East and West Africa had acquired plasma antibodies recognizing recombinant PF13_0006. The seropositive rate was slightly higher among the Gambian children, but this probably reflected that these children participated in a treatment trail and therefore had a recent exposure. The data suggest that RIFIN variants antigenically similar to the variable domain of PF13_0006 occur in the African parasite populations possibly in particular the unique sequence motif of the B1 RIFINs. These antibody responses were more common among older children than in adults. A similar decline in anti-PfEMP1 seropositivity was reported previously [46] and probably reflect that the antibody levels to these polymorphic proteins are short lived and that adults who have acquired partial immunity have less parasite exposure than the children. The prevalence of antibodies did not vary consistently with the level of transmission and was higher in the Tanzanian samples collected in 2008 than in the previous years, despite a general decline in transmission in the area [43]. This could reflect temporal shifts in the relative abundance of parasites carrying RIFIN variants closely related to PF13_0006, as similar fluctuations related to time and geographical location have been observed for antibody recognition of different PfEMP1 variants [47].

Confocal imaging of live, non-fixed, non-permeabilized free merozoites is challenging due to the small size and motility of these stages. However, present data does indicate that PF13_0006 is co-localized with MSP1 on the merozoite surface. This would place RIFIN along with the other variant surface antigens such as SURFINs [48] and STEVOR [3] which recently have been shown to be expressed on the surface of free merozoites. These studies of diverse multigene families, taken together, suggest that the antigens they encode play a role in evading the immune response against merozoites. However, as group B RIFIN only comprise ~25% (40 genes in the 3D7 genome) of the family, and the B1 sub-group around half of this proportion [22], indicating that there is scope for other members of the family to play different roles in the parasite life cycle. It is thus tempting to speculate that the B or B1 type RIFINs have become functionally specialized for a particular role in relation to merozoite egress or erythrocyte invasion.

Similarly, the PF13_0006 B1 RIFIN may serve a particular role either within the developing gametocyte, or in emergent gametes. Whether PF13_0006 serves a role in fertilization, e.g. locating and/or binding to a “partner” cell of opposite mating type, remains to be investigated. However, it is important to note that 3D7 sporozoites have also been found to specifically transcribe the PF13_0006 *rif* gene [22]. Extrapolating observations from single parasite isolates is unsound and there is a need to investigate *rif* expression on other genetic backgrounds, but the finding that PF13_0006 appear to be expressed in all free-living stages of 3D7 highlights the need for functional studies of this RIFIN in extracellular forms of the parasite.

The *rif* gene family is part of the *pir* (*Plasmodium* interspersed repeats) gene super-family of six variant multigene families found in *Plasmodium vivax* (*vir*), *Plasmodium ovale (oir), Plasmodium knowlesi* (*kir*) and in three rodent malarias (*Plasmodium chabaudi*, *cir*; *Plasmodium berghei*, *bir*; *Plasmodium yoelii*, *yir*). Sequence analysis of these gene families, as for *rif* genes, show compartmentalization into sub-groups/types indicative of specialized functions rather than sequence variation for antigenic variation alone [49]. This function may be common to all *Plasmodium* species which share life cycle, the common challenges of host immunity, and the need for invasion and rupture of host cells. The variable domain of PF13_0006, the domain to which the antibody was raised, seems to be semi-conserved in the C-terminal part for the B1 subtype RIFINs. This part of the protein may serve a common function for the subtype and it may be possible to generate antibodies targeting B1 RIFINs expressed by different parasite clones.

## Conclusions

In conclusion, the PF13_0006 B1 type RIFIN seem expressed on the surface of extra-cellular merozoites and gametes, which suggest some specific, common function for the B1 type RIFIN in *P. falciparum*’s extracellular forms in both the human host and mosquito vector. Further studies may demonstrate the functionality of this antigen family.

## Competing interests

The authors declare that they have no competing interests.

## Authors’ contributions

SBM, CWW carried out molecular biology studies, analysed data and wrote the paper. CJS, TL and TGT participated in the design, coordination and analysis of the study and helped to draft the manuscript. LT designed and carried out molecular biology studies, and analysed data. DCB and BD carried out molecular biology studies and revised the manuscript. DEA and JPL participated in the coordination of the study and revised the manuscript. All authors read and approved the final manuscript.

## Supplementary Material

Additional file 1 **Immunofluorescence analysis of PF13_0006 and PFD0070c expression in live parasites of the IT/FCR3 line.** Live Merozoites (first row) and schizonts (second row) of the IT/FCR3 parasite line were analyzed by staining with anti-PF13_0006 IgG (green), control IgG (green) and anti-MSP-1 Ab (red) in single staining experiments. Nuclei were stained with DAPI (blue). DIC shadow-cast images with the fluorescence image superimposed. Scale bar 5 μM. Click here for file

Additional file 2 **Western blot for PF13_0006 and PFD0070c expression in asexual parasite stages.** Protein extracts were obtained from 3D7 schizonts (3D7 S), 3D7 rings (3D7 R) and FCR3 schizonts (FCR3 S), and the expression of PF13_0006 RIFIN was analyzed by western blot. A band of approximately 39 kDa (circle) was detected by anti-PF13_0006 IgG in the 3D7 schizont extract. This is approximately similar size to that of the expected full length PF13_0006 protein, 37.8 kDa. Two additional bands of approximate size 26 and 51 kDa were also detected in 3D7 schizonts. No band of the expected size was detected in the 3D7 ring and FCR3 schizont extract. Two bands of approximately 27 and 78 kDa were observed with the control IgG in the 3D7 schizont extract. Click here for file

Additional file 3 **Transcript level fold differences of *rif *genes in the asexual ring and schizont stages of 3D7 line parasites.** Transcript fold changes of *rif* gene PF13_0006 and subgroups *RifA1*, *RifA2* and *RifA3*[22] between ring and schizont stage parasites, calculated by the ΔΔCt method. Click here for file

Additional file 4 **Immunofluorescence analysis of PF13_0006 expression in live asexual *Plasmodium falciparum *parasites.** Live schizonts (row one to three) and merozoites (row three to five) of the 3D7 parasite line were analyzed by staining with anti-PF13_0006 IgG (green) and anti-MSP-1 Ab (red). Nuclei were stained with DAPI (blue). The third row shows both schizonts and merozoites as some schizonts ruptured prior to imaging. DIC shadow-cast images with the fluorescence image superimposed in the first four coloumns and the fluorescence image (FI) alone in the last coloumn to augment the visualisation of the staining. Scale bar 5 μM. Click here for file

Additional file 5 **Expression of PF13_0006 on the surface of activated gametocytes (gametes).** Activated gametocytes (emerging gametes) of the *P. falciparum* 3D7 line were fixed but not permeabilized and stained with anti-PF13_0006 IgG (green) and anti-glycophorin A (red). Nuclei were stained with DAPI (blue). DIC shadow-cast images with the fluorescence image superimposed in the first four coloumns and the fluorescence image (FI) alone in the last coloumn. Scale bar 5 μM. Click here for file

Additional file 6 **Sequence alignments and conservation logo of the variable, V2, domain of RIFINs.** (A) The V2 domain of 481 3D7, IT/FCR3, HB3 and DD2 RIFIN sequences represented by three group A (PF10_0004, PFA0760w, PFE0020c), B (PFC1100w, PF11_0515, PF14_0005), B1 (PF13_0006, PFI0025c, PF10_0397), and B2 (PF07_0136, PFA0030c, PFI1810w) rifins, respectively. The V2 domain was split in three, V2-A, V2-B and V2-C, based on the two central cysteines marked with a star (*) and (B) Neighbour Joining Distance trees built from the amino acid muscle alignment of the three individual V2-sub-domains using the Poisson correction/NJ method. Red squares: RIFINB1; Green squares: RIFINB2; Black squares: RIFINB. The scale bar represents the proportion of different amino acids compared. (C) Sequence conservation logo for the V2-C domain of 37 B1 RIFIN sequences (19 3D7, eight IT/FCR3, five HB3, and five DD2, respectively) with the C-terminal part highlighted. The height of each position in the logos indicates the amino acid conservation level, and the height of the individual amino acids reflects their relative frequencies on the position and hence their contribution to the conservation. Hydrophobic amino acids: black; polar amino acids: green; acidic: red; basic: blue; neutrally charged: purple. Click here for file

Additional file 7 **IgG reactivity to recombinant PF13_0006 in plasma samples from individuals living in malaria endemic areas.** The anti-PF13_0006 IgG level in in age stratified plasma samples from (A) 1303 Tanzanian individuals and (B) 182 Gambian children. The IgG response was measured by the bead-based technology and data show median fluorescent intensity (MFI). Cut-off was based on the mean reactivity +2 SD of unexposed control donors and represented by the dashed line. Click here for file
