# Supplementary figures and images for: Expression of a type B RIFIN in Plasmodium falciparum merozoites and gametes
Source: Malar J. 2012 Dec 21;11:429. doi: 10.1186/1475-2875-11-429 (PMC3544569; doi:10.1186/1475-2875-11-429)

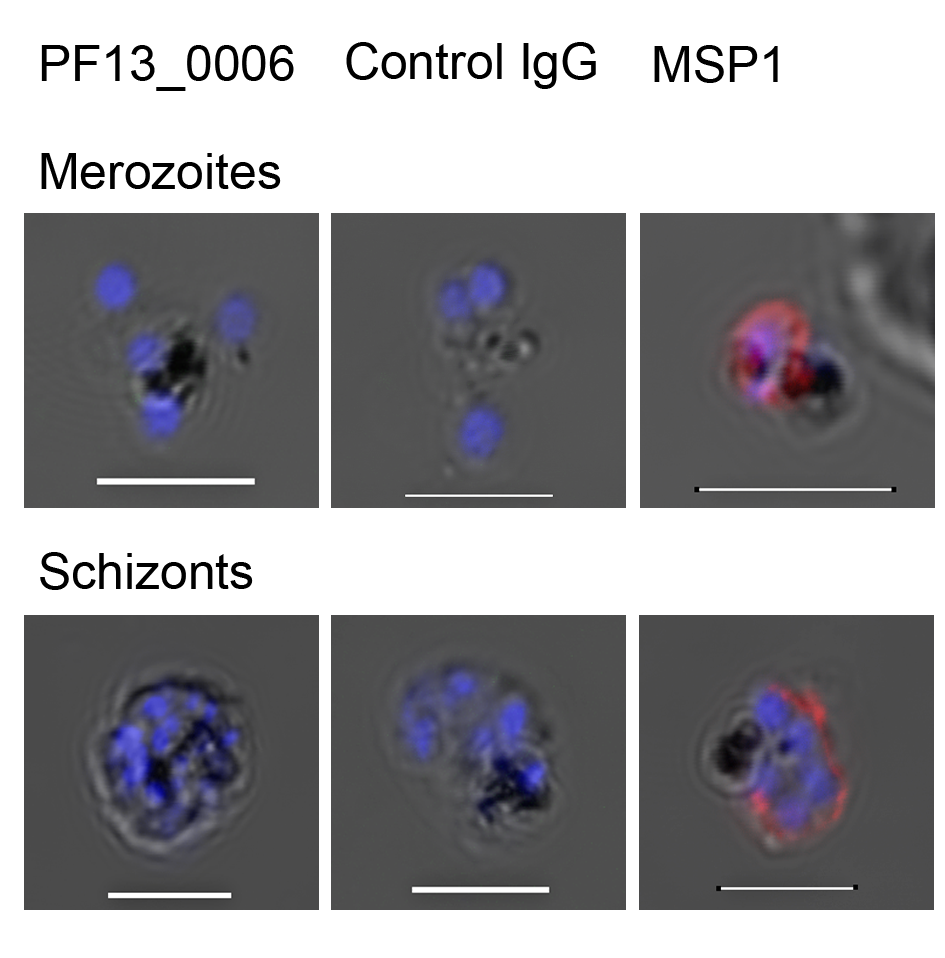

Supplement: Additional file 1 — Immunofluorescence analysis of PF13_0006 and PFD0070c expression in live parasites of the IT/FCR3 line. Live Merozoites (first row) and schizonts (second row) of the IT/FCR3 parasite line were analyzed by staining with anti-PF13_0006 IgG (green), control IgG (green) and anti-MSP-1 Ab (red) in single staining experiments. Nuclei were stained with DAPI (blue). DIC shadow-cast images with the fluorescence image superimposed. Scale bar 5 μM. [file 1475-2875-11-429-S1.tiff]

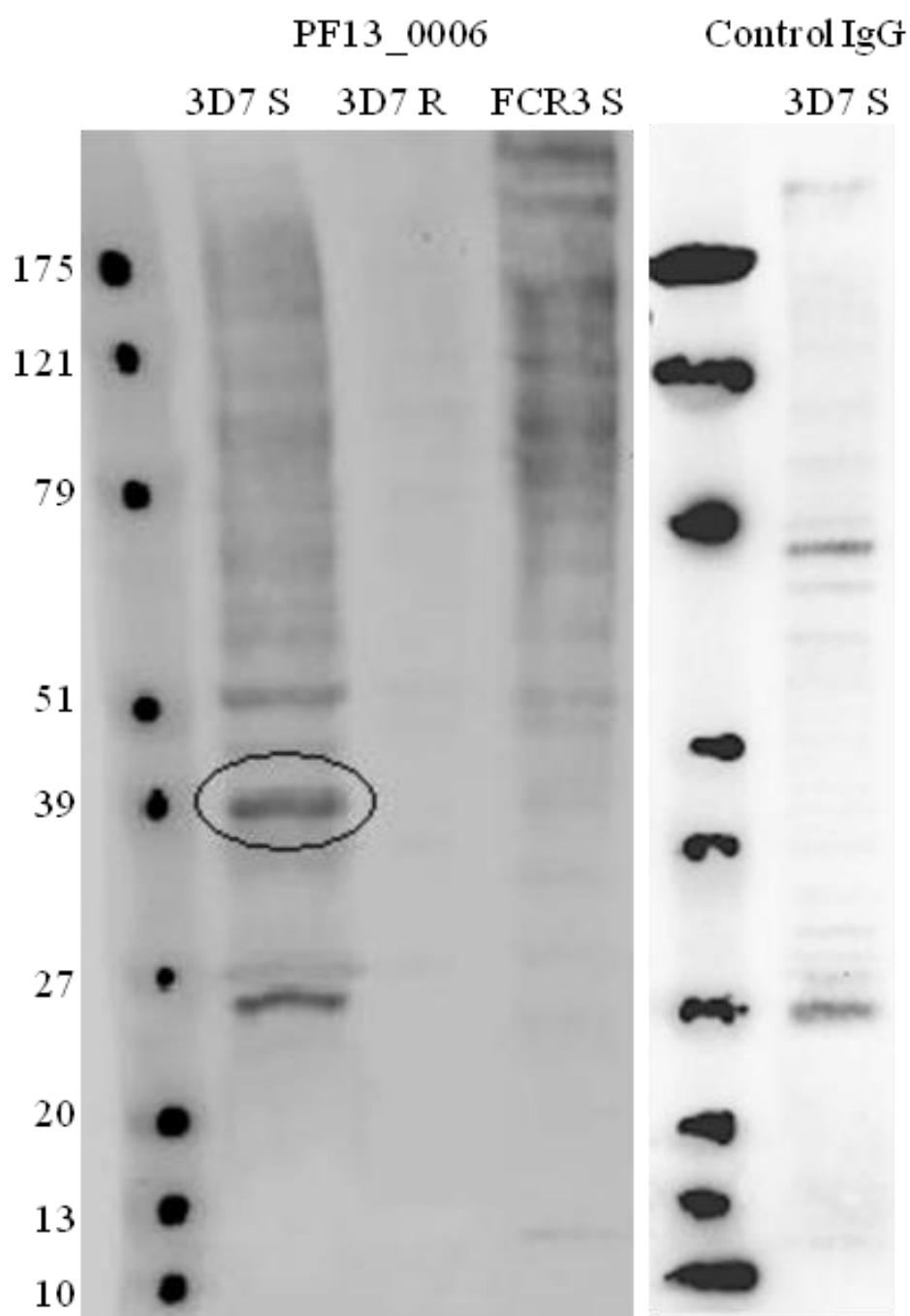

Supplement: Additional file 2 — Western blot for PF13_0006 and PFD0070c expression in asexual parasite stages. Protein extracts were obtained from 3D7 schizonts (3D7 S), 3D7 rings (3D7 R) and FCR3 schizonts (FCR3 S), and the expression of PF13_0006 RIFIN was analyzed by western blot. A band of approximately 39 kDa (circle) was detected by anti-PF13_0006 IgG in the 3D7 schizont extract. This is approximately similar size to that of the expected full length PF13_0006 protein, 37.8 kDa. Two additional bands of approximate size 26 and 51 kDa were also detected in 3D7 schizonts. No band of the expected size was detected in the 3D7 ring and FCR3 schizont extract. Two bands of approximately 27 and 78 kDa were observed with the control IgG in the 3D7 schizont extract. [file 1475-2875-11-429-S2.pdf]

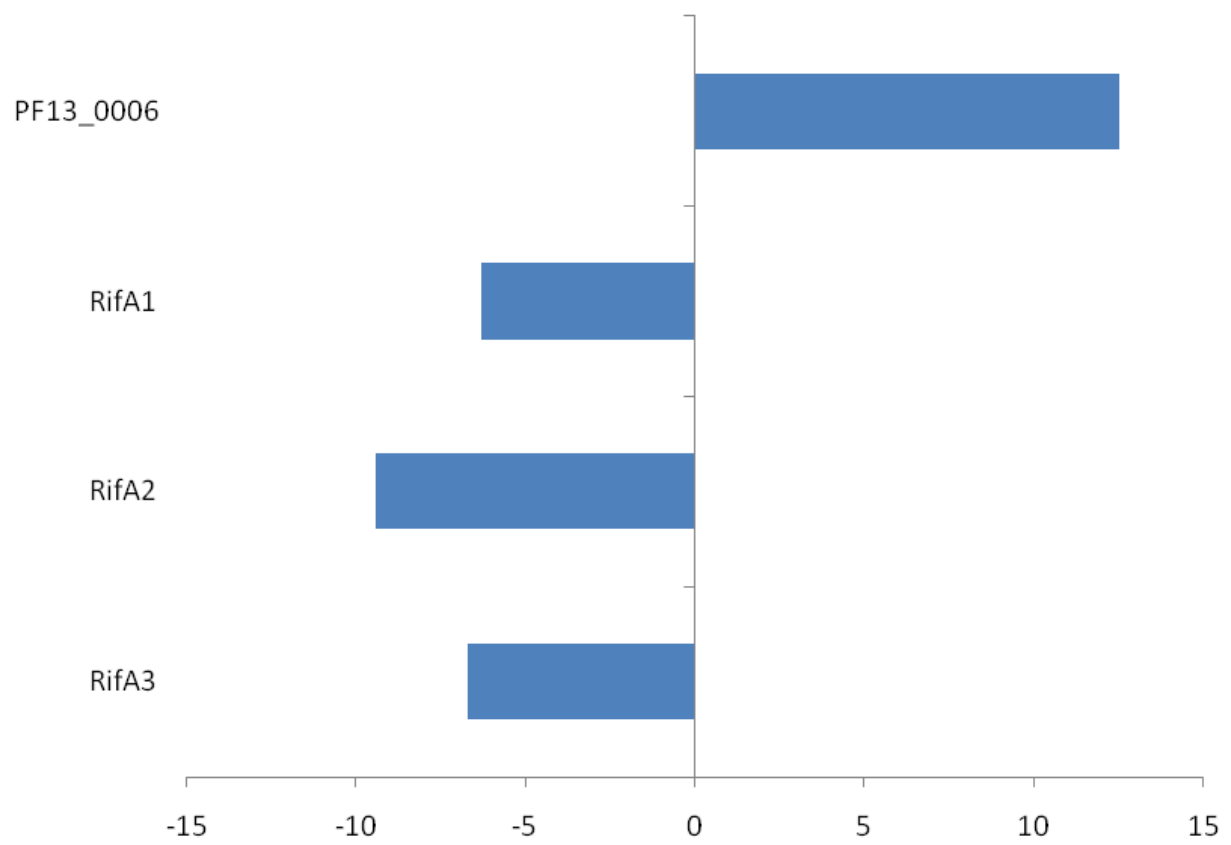

Supplement: Additional file 3 — Transcript level fold differences of rif genes in the asexual ring and schizont stages of 3D7 line parasites. Transcript fold changes of rif gene PF13_0006 and subgroups RifA1, RifA2 and RifA3[22] between ring and schizont stage parasites, calculated by the ΔΔCt method. [file 1475-2875-11-429-S3.pdf]

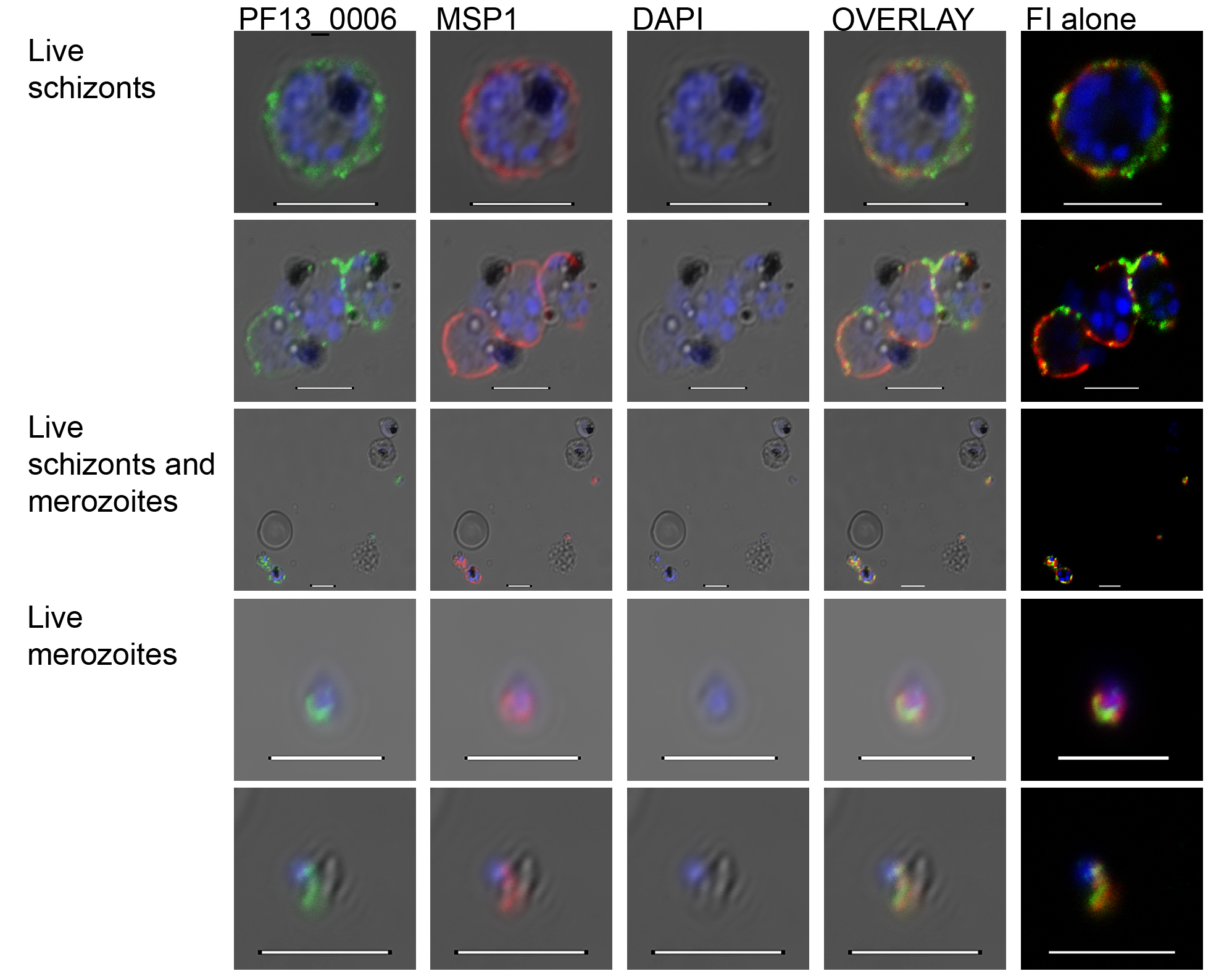

Supplement: Additional file 4 — Immunofluorescence analysis of PF13_0006 expression in live asexual Plasmodium falciparum parasites. Live schizonts (row one to three) and merozoites (row three to five) of the 3D7 parasite line were analyzed by staining with anti-PF13_0006 IgG (green) and anti-MSP-1 Ab (red). Nuclei were stained with DAPI (blue). The third row shows both schizonts and merozoites as some schizonts ruptured prior to imaging. DIC shadow-cast images with the fluorescence image superimposed in the first four coloumns and the fluorescence image (FI) alone in the last coloumn to augment the visualisation of the staining. Scale bar 5 μM. [file 1475-2875-11-429-S4.tiff]

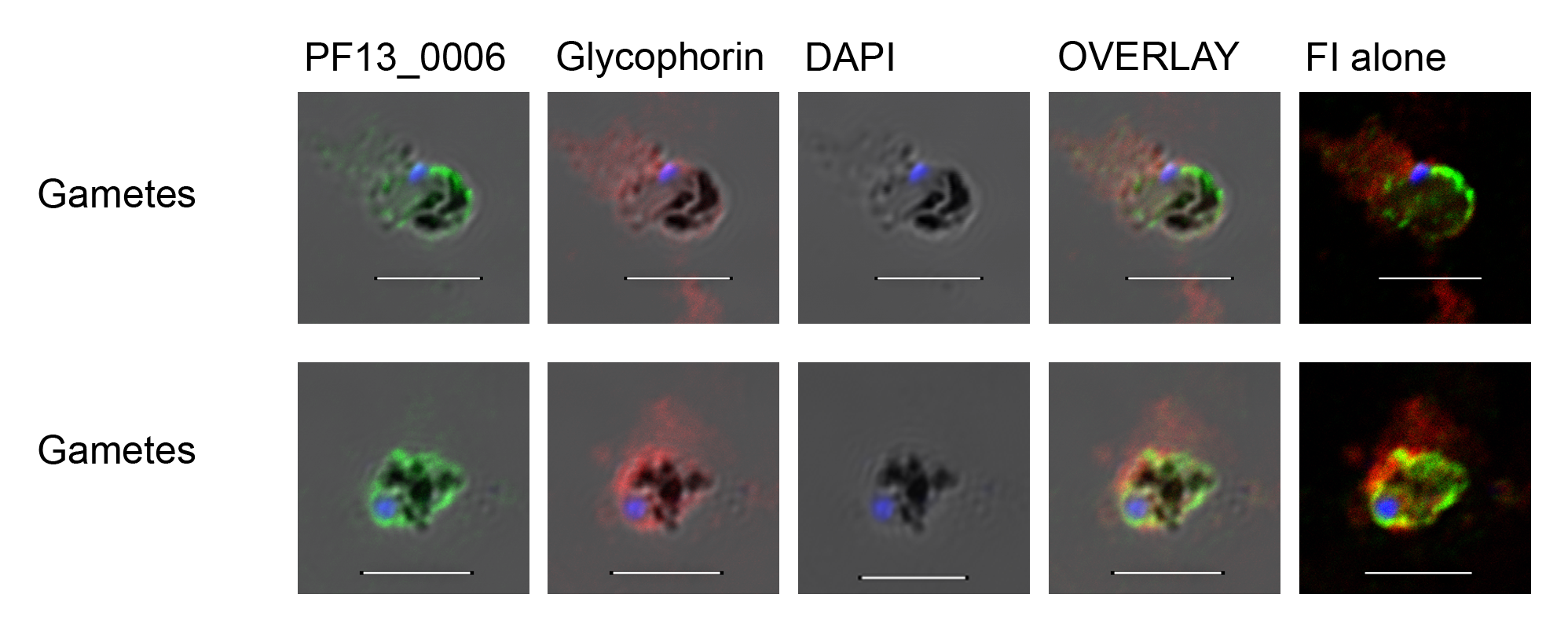

Supplement: Additional file 5 — Expression of PF13_0006 on the surface of activated gametocytes (gametes). Activated gametocytes (emerging gametes) of the P. falciparum 3D7 line were fixed but not permeabilized and stained with anti-PF13_0006 IgG (green) and anti-glycophorin A (red). Nuclei were stained with DAPI (blue). DIC shadow-cast images with the fluorescence image superimposed in the first four coloumns and the fluorescence image (FI) alone in the last coloumn. Scale bar 5 μM. [file 1475-2875-11-429-S5.tiff]

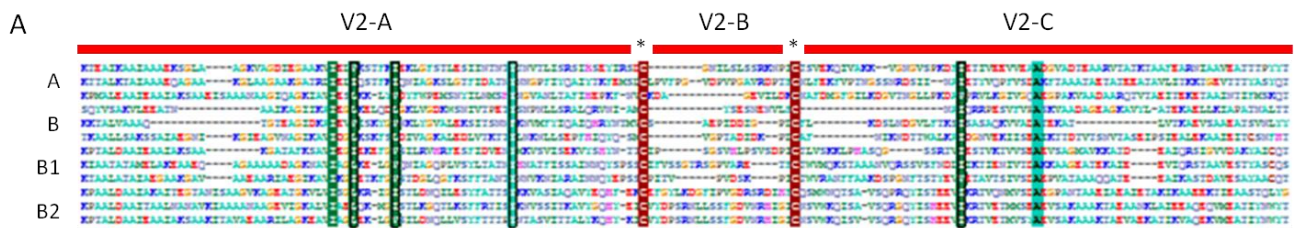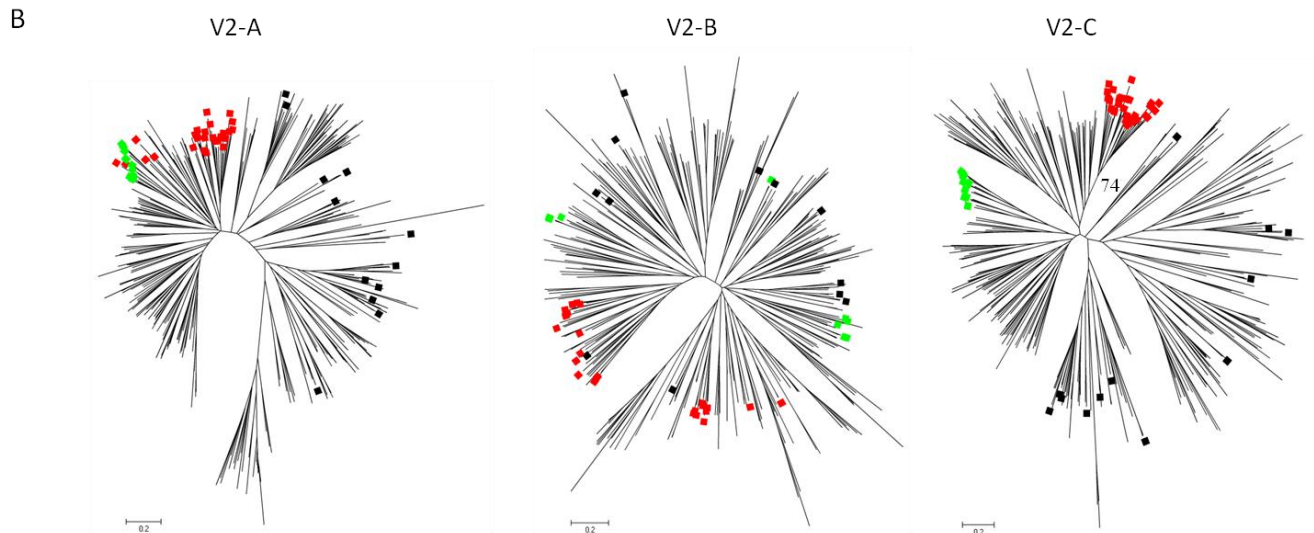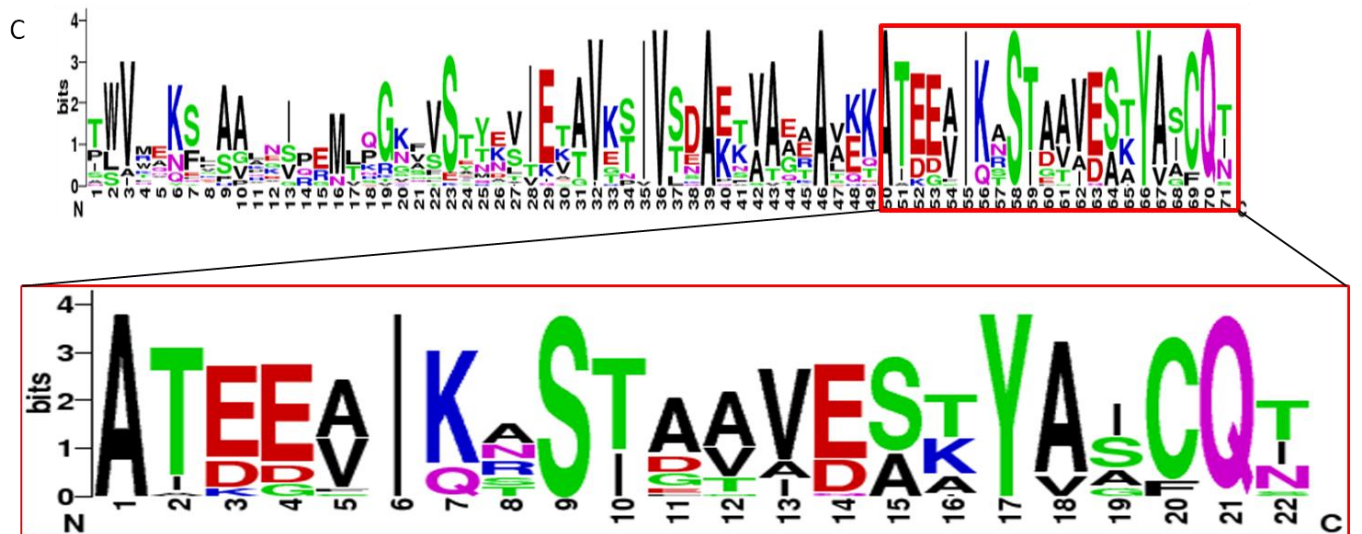

Supplement: Additional file 6 — Sequence alignments and conservation logo of the variable, V2, domain of RIFINs. (A) The V2 domain of 481 3D7, IT/FCR3, HB3 and DD2 RIFIN sequences represented by three group A (PF10_0004, PFA0760w, PFE0020c), B (PFC1100w, PF11_0515, PF14_0005), B1 (PF13_0006, PFI0025c, PF10_0397), and B2 (PF07_0136, PFA0030c, PFI1810w) rifins, respectively. The V2 domain was split in three, V2-A, V2-B and V2-C, based on the two central cysteines marked with a star (*) and (B) Neighbour Joining Distance trees built from the amino acid muscle alignment of the three individual V2-sub-domains using the Poisson correction/NJ method. Red squares: RIFINB1; Green squares: RIFINB2; Black squares: RIFINB. The scale bar represents the proportion of different amino acids compared. (C) Sequence conservation logo for the V2-C domain of 37 B1 RIFIN sequences (19 3D7, eight IT/FCR3, five HB3, and five DD2, respectively) with the C-terminal part highlighted. The height of each position in the logos indicates the amino acid conservation level, and the height of the individual amino acids reflects their relative frequencies on the position and hence their contribution to the conservation. Hydrophobic amino acids: black; polar amino acids: green; acidic: red; basic: blue; neutrally charged: purple. [file 1475-2875-11-429-S6.pdf]

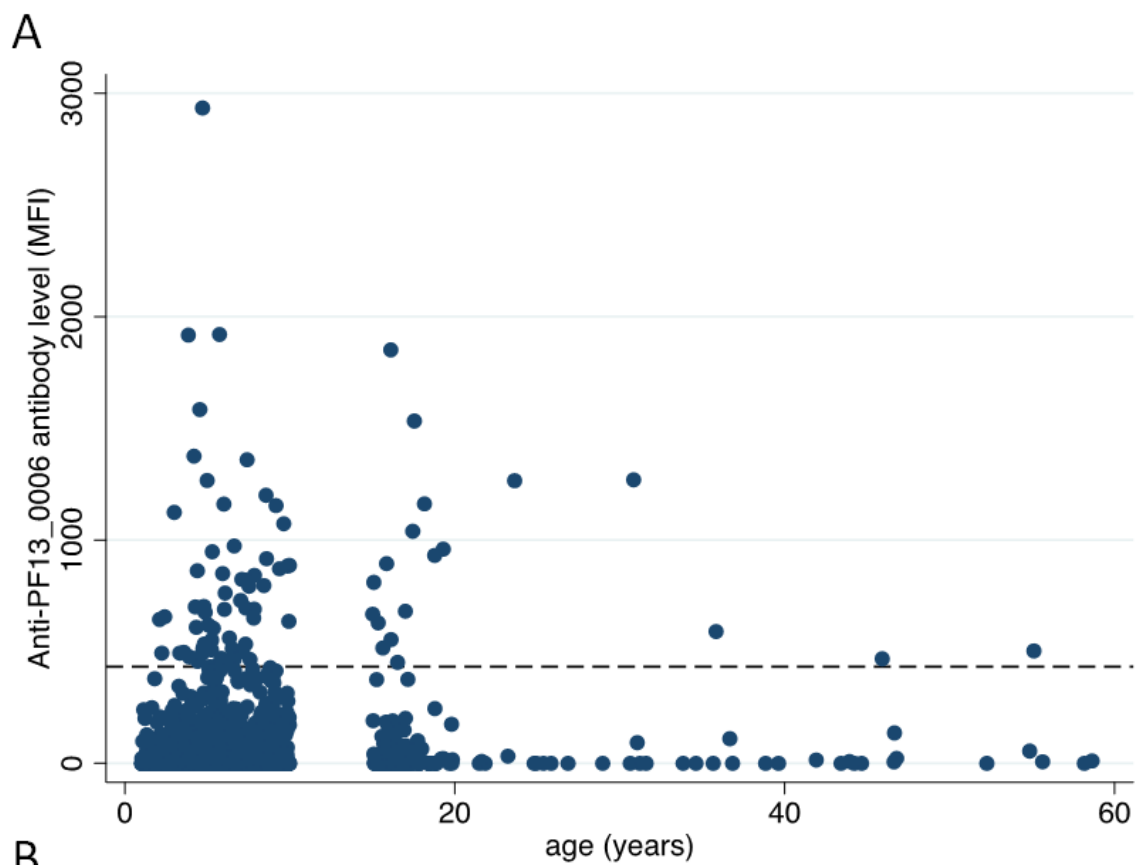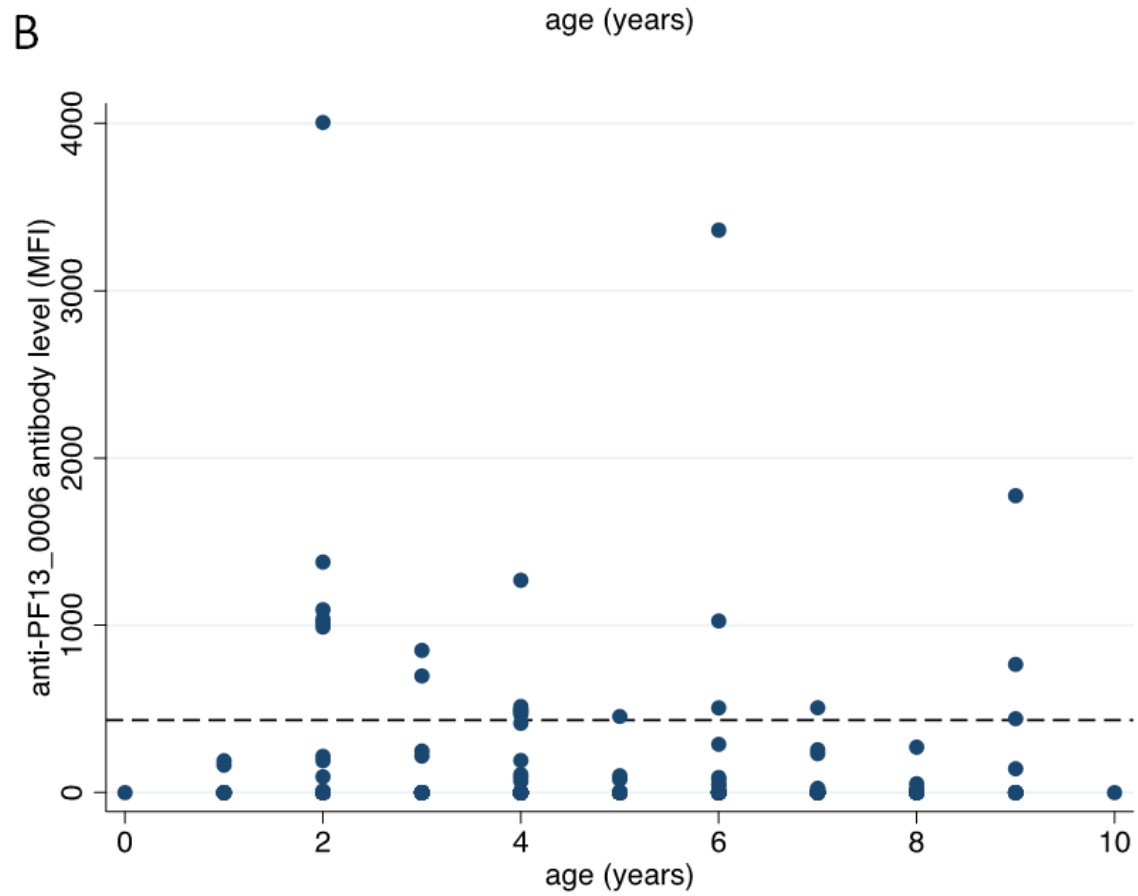

Supplement: Additional file 7 — IgG reactivity to recombinant PF13_0006 in plasma samples from individuals living in malaria endemic areas. The anti-PF13_0006 IgG level in in age stratified plasma samples from (A) 1303 Tanzanian individuals and (B) 182 Gambian children. The IgG response was measured by the bead-based technology and data show median fluorescent intensity (MFI). Cut-off was based on the mean reactivity +2 SD of unexposed control donors and represented by the dashed line. [file 1475-2875-11-429-S7.pdf]
